# TTSNet: Transformer–Temporal Convolutional Network–Self-Attention with Feature Fusion for Prediction of Remaining Useful Life of Aircraft Engines

**DOI:** 10.3390/s25020432

**Published:** 2025-01-13

**Authors:** Zhaofei Li, Shilin Luo, Haiqing Liu, Chaobin Tang, Jianguo Miao

**Affiliations:** 1 School of Automation and Information Engineering, Sichuan University of Science & Engineering, Yibin 644000, China; lzf825@suse.edu.cn (Z.L.); 323085404212@stu.suse.edu.cn (H.L.); 324085404513@stu.suse.edu.cn (C.T.); 2 Key Laboratory of Artificial Intelligence of Sichuan Province, Yibin 644000, China; miaojg@cqupt.edu.cn; 3 School of Automation, Chongqing University of Posts and Telecommunications, Chongqing 400065, China

**Keywords:** TCN, transformer, self-attention, aircraft engines, RUL prediction

## Abstract

Accurately predicting the remaining useful life (RUL) is crucial for ensuring the safety and reliability of aircraft engine operation. However, aircraft engines operate in harsh conditions, with the characteristics of high speed, high temperature, and high load, resulting in high-dimensional and noisy data. This makes feature extraction inadequate, leading to low accuracy in the prediction of the RUL of aircraft engines. To address this issue, Transformer-TCN-Self-attention network (TTSNet) with feature fusion, as a parallel three-branch network, is proposed for predicting the RUL of aircraft engines. The model first applies exponential smoothing to smooth the data and suppress noise to the original signal, followed by normalization. Then, it uses a parallel transformer encoder, temporal convolutional network (TCN), and multi-head attention three-branch network to capture both global and local features of the time series. The model further completes feature dimension weight allocation and fusion through a multi-head self-attention mechanism, emphasizing the contribution of different features to the model. Subsequently, it fuses the three parts of features through a linear layer and concatenation. Finally, a fully connected layer is used to establish the mapping relationship between the feature matrix and the RUL label, obtaining the RUL prediction value. The model was validated on the C-MAPSS aircraft engine dataset. Experimental results show that compared to other related RUL models, the RMSE and Score reached 11.02 and 194.6 on dataset FD001, respectively; on dataset FD002, the RMSE and Score reached 13.25 and 874.1, respectively. On dataset FD003, the RMSE and Score reached 11.06 and 200.1 and on dataset FD004, the RMSE and Score reached 18.26 and 1968.5, respectively, demonstrating better performance of RUL prediction.

## 1. Introduction

The aircraft engines are the core component of an aircraft and their health status directly influences the safety of flight operations [1]. The prediction of RUL is one of the cores of Prognostics and Health Management (PHM) technology, which not only forecasts the future health status of the engines but also provides reasonable maintenance strategies for their health management [2]. The maintenance costs of aircraft engines are high. Premature maintenance can result in economic losses, while delayed maintenance can lead to accidents. Therefore, in order to ensure the safety and reliability of aero-engine operations, it is crucial to accurately predict the engines’ RUL.

The methods of RUL prediction can be divided into knowledge-based approaches, physical model-based approaches, and data-driven approaches [3]. Knowledge-based models predict RUL by knowledge and experience, providing interpretable results. However, it is challenging to obtain accurate knowledge from experience and the sources of knowledge are limited [3]. When the system complexity is low, physical model-based methods can establish accurate mathematical and physical models of the engines to predict their RUL, with high prediction accuracy [4]. The drawback of this approach is that the internal structure, operational mechanisms, and degradation principles of the engines need to be understood. Due to the fact that aero-engines have a complex structure and numerous components, it is challenging to develop accurate models and the precision of life prediction can be influenced.

In contrast, data-driven methods do not require in-depth knowledge of the device’s degradation mechanisms [5], but only need the internal state data of the equipment. Through machine learning methods, large amounts of historical data can be utilized to establish models for RUL prediction. RUL prediction methods based on data are mainly divided into shallow model-based approaches and deep learning-based approaches [6]. There has been considerable research on RUL based on shallow models, such as Khelif et al. [7], who used support vector regression to establish the relationship between sensor values and health indicators, estimating RUL at any point in the degradation process. Chen et al. [8] introduced a life prediction method using Lasso feature selection and random forest regression. While RUL methods based on shallow models have achieved certain results, it is difficult for their predictive performance to significantly improve beyond a certain stage due to the limitations of the learning capabilities of shallow models.

In recent years, with the continuous development of deep learning technology, it has become the mainstream and cutting-edge method in the field of RUL prediction due to its superior predictive performance. Heimes [9] used recurrent neural networks (RNNs) to predict the RUL of aircraft engines. However, it is difficult for RNNs to effectively capture long-term dependencies in processing long sequence data due to their insufficient memory capacity. To address this issue, researchers have proposed variants of RNNs such as Long Short-Term Memory networks (LSTM) and Gated Recurrent Units (GRUs). For instance, Li et al. [10] employed principal component analysis for dimensionality reduction to obtain the correlation of sensor data, followed by using LSTM to extract temporal features for predicting the RUL of aero-engines. Boujamz et al. [11] introduced an attention mechanism-enhanced LSTM method, improving the prediction capability of engines. Qin et al. [12] proposed a gated recurrent neural network with attention gates for the prediction of the RUL of bearing. To obtain capabilities of more robust feature extraction, researchers have utilized autoencoders in RUL prediction. For example, Fan et al. [13] first used a bidirectional LSTM autoencoder to extract basic features and then further predicted RUL through a transformer encoder. Ren et al. [14] used a deep autoencoder and deep neural network to achieve a good RUL prediction efficiency of bearing. On the other hand, to fully extract RUL features, researchers have also utilized convolutional neural network (CNN) methods in RUL prediction. Li et al. [15] constructed a deep CNN with convolutional kernels of different size for feature extraction to predict the RUL of engines. Lan et al. [16] proposed an aero-engine RUL method combining attention mechanisms with residual deep separable convolutional networks. Che et al. [17] used 1D convolutional network for degradation regression analysis, followed by a Bidirectional Long Short-Term Memory (Bi-LSTM) neural network for time series prediction, obtaining future degradation trends. Zhang [18] employed CNN for feature extraction, Bi-LSTM for capturing long-term dependencies in features and attention mechanisms to highlight important parts of features, enhancing the prediction accuracy of the model. RNNs and their variants have been widely applied in RUL prediction, but they use the last time node for feature prediction, ignoring the feature information from other historical nodes. Moreover, they cannot perform parallel inference and have poor interpretability. One-dimensional convolutional neural networks require that the time series is segmented into fixed-size time windows, necessitating significant computational resources and large amounts of data for RUL prediction, and are prone to overfitting. In contrast, the transformer model can perform parallel computations on input data, calculating global dependencies across the entire input sequence through self-attention, thereby capturing more comprehensive contextual information [19]. Zhou et al. [20] used transformer to mine the mapping relationship between features and RUL for the RUL prediction of bearing. Zhang et al. [21] employed dual encoders to extract respectively features of different sensors and time steps, making it more effective for processing long data sequences. Ma et al. [22] proposed a multi-encoder feature fusion method using transformer, selecting input data with two different time lengths, using permutation entropy to analyze relationships among sensors, independently extracting features from operational condition data. Zhang et al. [23] introduced an adaptive optimal lightweight transformer with pruning to reduce model redundancy and improve prediction accuracy. Additionally, some researchers have adopted methods based on patch-segmented transformer models. For instance, Fan et al. [24] utilized a hierarchical encoder–decoder structure to integrate multi-scale information, achieving hierarchical prediction. Ren et al. [25] proposed a dynamic length transformer that can adaptively learn sequence representations of varying lengths. Mamba is built on state space models, which can selectively process time series input information and is simpler in structure. It has also been applied to RUL prediction. For example, Shi [26] proposed MambaLithium to capture the complex aging and charging dynamics of lithium-ion batteries, with advantages in terms of predicting battery health.

The transformer can capture global features of time series but is insensitive to local information. Aero-engines operate in high-speed, high-load, high-temperature, and harsh environments, where their operational data are characterized by high noise, high dimensionality, and indistinct states. Local information can significantly impact the accuracy of life prediction models. For multi-dimensional time series, the transformer processes variables of different dimensions equally, but these variables contain varying amounts of degradation information. TCN [27] is a stable network that can parallelly compute data across all time steps, with strong long-term dependency modeling capabilities and fewer parameters. Zhang et al. [28] proposed an RUL prediction method combining TCN with attention, assigning different weights to different sensor features and time steps through attention mechanisms. However, for long time series, TCN can easily lose information and fixed-length convolutional kernels cannot flexibly handle time series of varying lengths. In some cases, the transformer may also fail to effectively capture sparse features in the data.

Therefore, leveraging the strengths of both models and the advantages of weight assignment of multi-head self-attention mechanisms to important features, this study presents a model for the RUL prediction of aero-engines, which fuses features from a three-branch network of a transformer, TCN, and multi-head self-attention mechanism, abbreviated as TTSNet. The model first uses exponential smoothing to reduce noise, then captures both global degradation information and detailed features based on a three-branch network of transformer encoders, TCN, and multi-head attention and highlights the contribution of different features to the model. The three types of features are processed through linear layers and concatenated, followed by a fully connected layer to establish the mapping relationship between the feature matrix and the output RUL, obtaining the RUL prediction value and achieving the RUL prediction of aircraft engines.

## 2. The Structure of TTSNet

### 2.1. Temporal Convolutional Network

**Figure 1:**
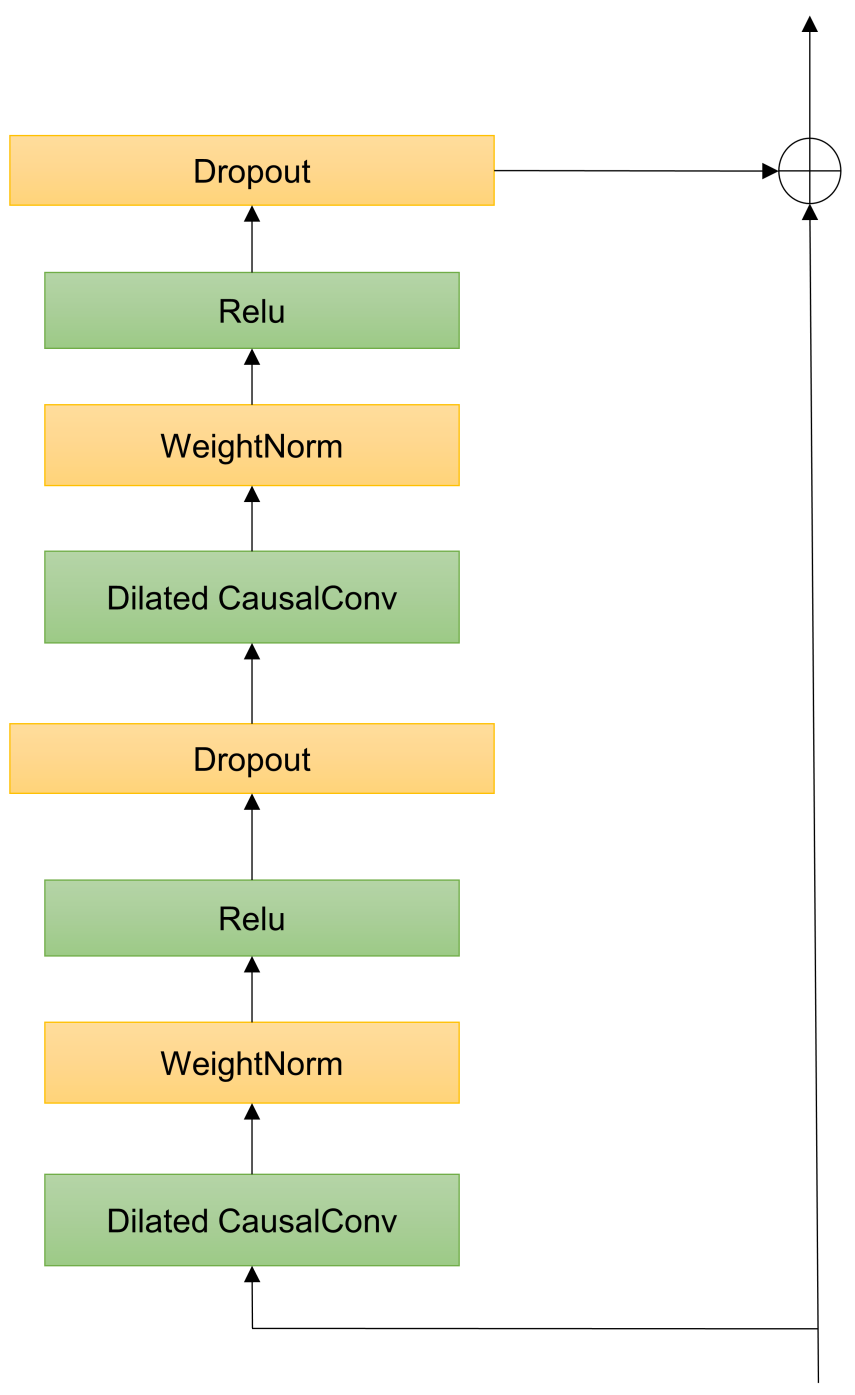
Structure of TCN.

TCN is a 1D deep convolutional network designed for processing time series data, capturing local feature information of the input sequence through one-dimensional convolutional kernels. TCN primarily consists of causal dilated convolutions and residual connections [27], as illustrated in Figure 1.

**Figure 2:**
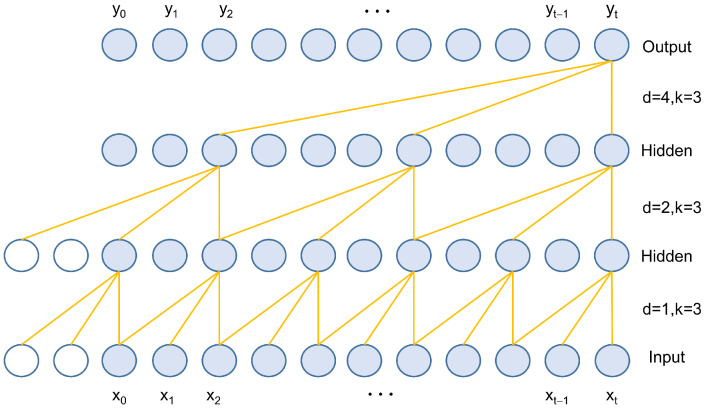
Structure of causal dilated convolution.

Time series data exhibit causal relationships, where the output at the current moment is determined only by the inputs from past moments and not by future data. The causal dilated convolution structure, shown in Figure 2, is primarily used to capture dependencies between features at the current and past moments, as depicted in Figure 2. The input to the nodes in the upper layer of the neural network originates from the corresponding nodes in the lower layer and their preceding positions. To achieve a larger receptive field and capture longer dependencies, numerous additional hidden layers are required between the output and input layers.

TCN introduces dilated convolutions, which expand the receptive field while reducing the number of hidden layers by adding gaps in the standard convolutional kernels. Dilated convolutions allow for input sampling at intervals, with the sampling rate controlled by the dilation factor d in Figure 2. When d = 1, each point in the input is sampled, making the dilated convolution identical to a standard convolution with a kernel size of 3. When d = 2, every second point is sampled as input, increasing the receptive field of each node to 5. The receptive field size of the dilated convolution in the upper layer is approximately double that of the previous layer. Consequently, dilated convolutions enable the effective window size to grow exponentially with the number of layers, allowing the convolutional network to achieve a large receptive field with relatively few layers.

By employing residual connections, TCN facilitates easier gradient propagation during training, accelerating convergence and enhancing performance. The convolutional layers in TCN have adjustable receptive fields and strides, allowing the network to learn different features of the input sequence at various levels. Through effective adjustment, TCN can more effectively learn local feature representations of the input.

### 2.2. Transformer Encoder Structure

**Figure 3:**
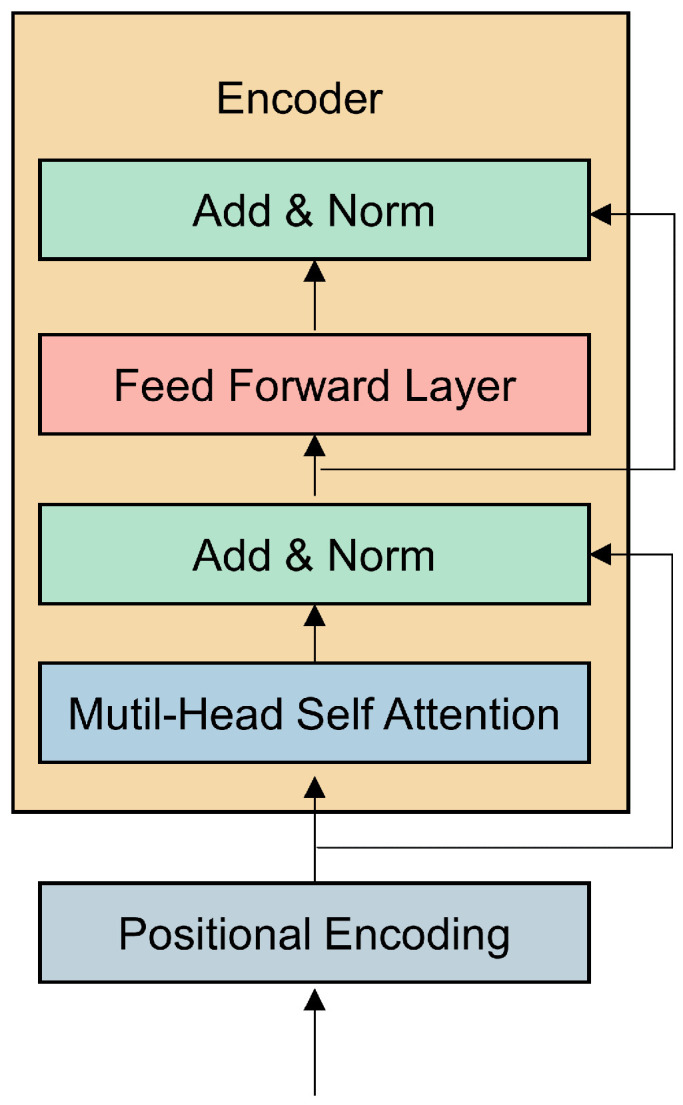
Structure of transformer encoder.

Transformer consists of an encoder and a decoder, initially designed for natural language translation [19]. Since the model in this paper requires extracting global degradation information from the data, only the encoder part is utilized. The encoder comprises positional encoding, multi-head attention layers, and fully connected feedforward layers, and its structure as illustrated in Figure 3.

Since the transformer does not inherently contain sequence position information, it requires positional encoding to express the order of the input sequence. Positional encoding uses sine and cosine functions to represent absolute positions, as shown in the following formula:

(1) $$P\left(k,2i\right)=\sin\left(k/10,000^{2i/d}\right)$$

(2) $$P\left(k,2i+1\right)=\cos\left(k/10,000^{2i/d}\right)$$

where *P* represents the location code, *k* represents the position of the current moment sequence in the input time series and *d* represents the feature dimension of a single sequence.

**Figure 4:**
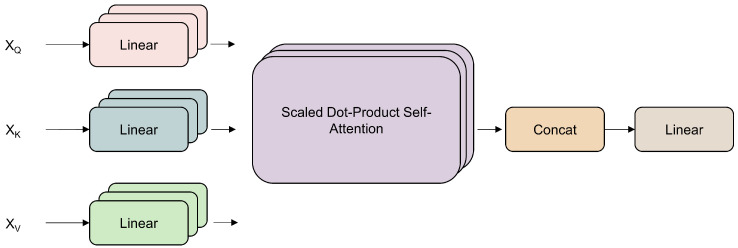
Structure of multi-head self-attention.

The structure of the multi-head attention of the encoder is shown in Figure 4. By taking the time series as input, the self-attention mechanism transforms the input sequence into vectors containing sequence feature information. The self-attention mechanism calculates the correlation between any two positions in the sequence, generating context-aware representations, addressing the problem of capturing long-range dependencies, and improving the efficiency of processing sequence data. The self-attention mechanism uses scaled dot-product to calculate the attention distribution: first, the dot-product of the key vector *K* and the query vector *Q* is computed to obtain the initial attention weights, then the softmax function is used to normalize these weights, and finally, the value vector *V* is multiplied by the normalized weights.

(3) $$Q=XW^{q}$$

(4) $$K=XW^{\dot{k}}$$

(5) $$V=XW^{\nu }$$

(6) $$Attention\left(Q,K,V\right)=soft\max\left(\frac{QK^{T}}{\sqrt{d_{k}}}\right)V$$

The feedforward layer of the encoder takes the output of the self-attention layer as input and enhances the model’s expressive capability through nonlinear transformations, with the activation function being ReLU, and its formula is as follows:

(7) $$FFN\left(x\right)=ReLU\left(xW_{1}+b_{1}\right)W_{2}+b_{2}$$

### 2.3. Structure of TTSNet Model

**Figure 5:**
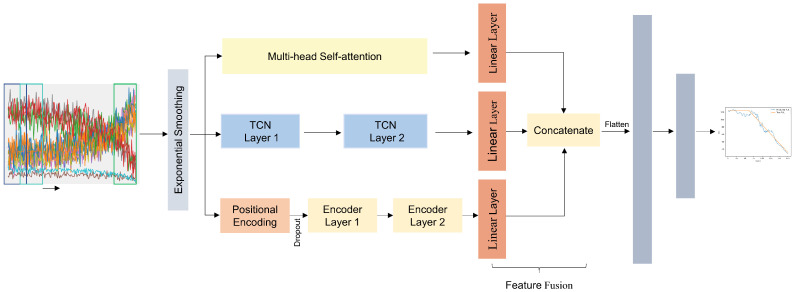
Structure of TTSNet.

The primary objective of this study is to enhance the accuracy of RUL prediction. To fully leverage the temporal and sensor information in the aero-engine data, a three-branch Transformer-TCN-Self-attention network with feature fusion for the life prediction of aero-engines is proposed. The structure of the model is shown in Figure 5. It mainly consists of exponential smoothing, multi-head self-attention, TCN layers, encoder layers, and fully connected layers. The model first applies exponential smoothing to the input data for smoothing; then, the entire input feature time series are processed in parallel through 2 layers temporal convolutional network to extract local degradation features. Each layer of the network uses a convolution kernel of size 3 and an activation function of ReLU; to fully extract global features, another branch uses 2 layers of transformer encoders in parallel to extract global degradation features from multivariate time series The number of attention heads in each encoder layer is 2, and the number of neurons in the feedforward layer is 28; to distinguish the contribution of different input sensor features to the prediction, the third branch employs multi-head self-attention to allocate weights across the sensor dimensions of the time series and the number of attention heads is 5; the outputs of the three parts are then fused through linear layers; finally, the feature map is expanded into a one-dimensional matrix, and a fully connected layer is used to establish the mapping relationship between the feature matrix and the RUL label, obtaining the RUL prediction value. The fully connected layer consists of 3 layers, with the number of neurons in each layer being 100, 50, and 1, respectively. The activation function for the first two layers is ReLU, and the last layer is a linear layer.

## 3. Introduction of C-MAPSS Dataset and Data Preprocessing

### 3.1. Introduction of C-MAPSS Dataset

The experiment utilized the C-MAPSS dataset from NASA for predicting the RUL of aircraft engines [29]. The C-MAPSS dataset includes 3 operational parameters, 21 sensor status monitoring parameters, and corresponding operational time cycle. Table 1 provides an overview of the C-MAPSS dataset, and Table 2 lists the sensor status monitoring parameters. The dataset is divided into four subsets: FD001, FD002, FD003 and FD004, each with training and test sets. The training sets record sensor monitoring data from the first operation to gradual failure under different fault modes and operating conditions for a certain type of turbofan engine, while the test sets record sensor monitoring data from the degradation point to gradual failure. The four subsets were used in the experiment to verify the model’s effectiveness. The following description is based on the FD001 dataset as an example.

### 3.2. Data Feature Selection

**Figure 6:**
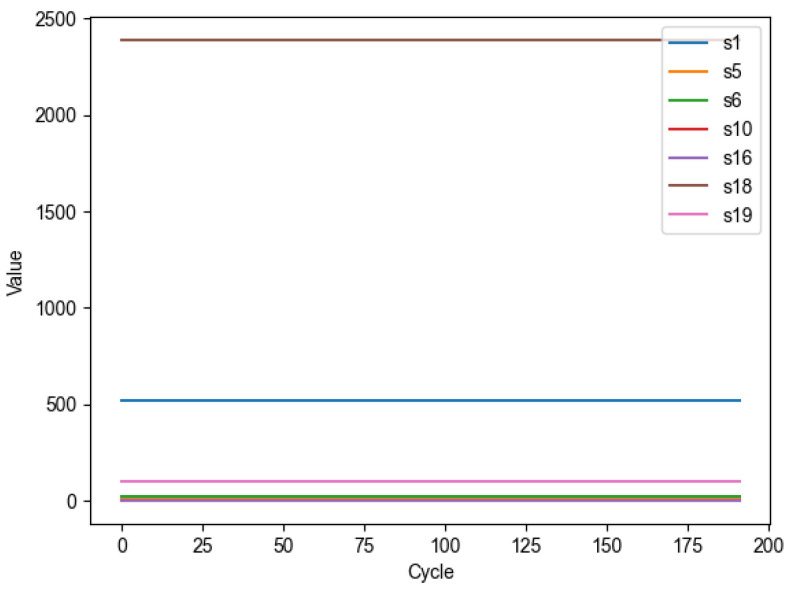
Seven invariant sensor data on FD001.

The FD001 subset of the C-MAPSS dataset contains a total of 21 sensor monitoring parameter status data. Although multidimensional features contain more degradation information of the engine, high-dimensional input features also increase the difficulty of engine life prediction, leading to inaccurate model predictions. Therefore, it is necessary to select features with more degradation information from the multivariate features and eliminate sensor variables with less information. In Figure 6, the horizontal axis represents the number of flight cycles, and the vertical axis represents the sequence number of the variables. From Figure 6, it can be seen that as the number of engine cycles increases, features such as Total temperature at fan inlet, Pressure at fan inlet, Total pressure in bypass-duct, Engine pressure ratio, Burner fuel–air ratio, Demanded fan speed, and Demanded corrected fan speed in the FD001 dataset show no significant change trends, with constant values, and have no impact on remaining life prediction. Therefore, these features are eliminated from the 21 features, and the remaining sensor status monitoring data, totaling 14 features, are selected as the model’s input.

### 3.3. Data Denoising and Normalization

**Figure 7:**
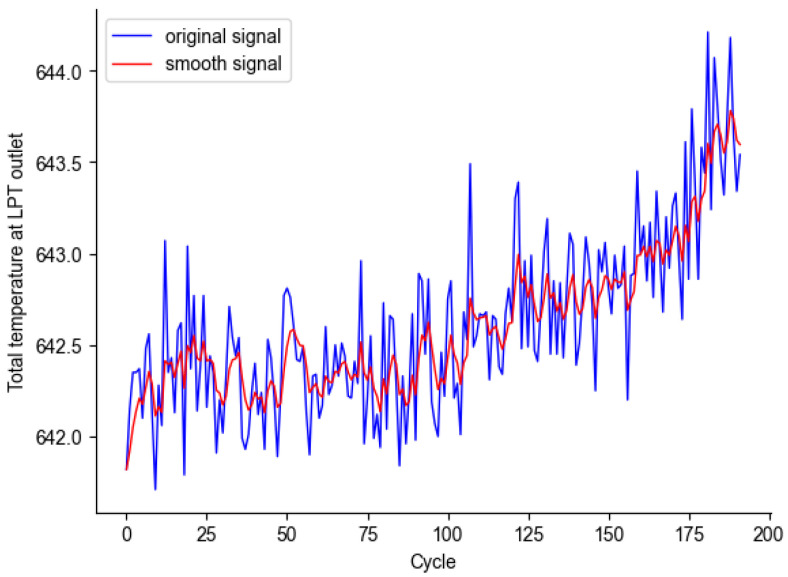
Exponential smoothing of the second engine on FD001.

The operation environment of aero-engines is complex, and the condition monitoring data is highly noisy, which can significantly affect the accuracy of the RUL model. Exponential smoothing can preserve the trend of the data, and by using a moving weighted average, it can minimize the differences in the time series [30]. Therefore, the experiment employs exponential smoothing to reduce the impact of noise on the prediction model, with the following formula:

(8) $$\left\{\begin{array}{c} y_{t}=\alpha \cdot x_{t}+\left(1-\alpha \right)\cdot x_{t-1},t\geq 2\\ y_{t}=x_{t},t=1 \end{array}\right.$$

where $x_{t}$ represents the sensor data value at time *t*, $y_{t}$ is the corresponding value after smoothing, and α is the smoothing coefficient, with a range of [0,1]. A larger value results in a more fluctuating curve, while a smaller value results in a smoother curve. To smooth the sensor signal while retaining degradation information as much as possible, α is set to 0.3. The smoothing effect on the Total temperature at LPT outlet of the second engine in FD001 is shown in Figure 7.

The data of sensors have different units. To eliminate the influence of dimensions and improve the accuracy and training time of the model, the min-max normalization method is used to normalize the data to the range [0,1], with the formula as follows:

(9) $$x_{\mathrm{new}}^{j}=\frac{x^{j}-x_{\min}^{j}}{x_{\max}^{j}-x_{\min}^{j}}$$

where *j* represents the model of the sensor, $x_{max}^{j}$ represents the maximum value in data of the *j*-th sensor, and $x_{min}^{j}$ represents the minimum value in data of the *j*-th sensor.

### 3.4. Time Window Sampling

**Figure 8:**
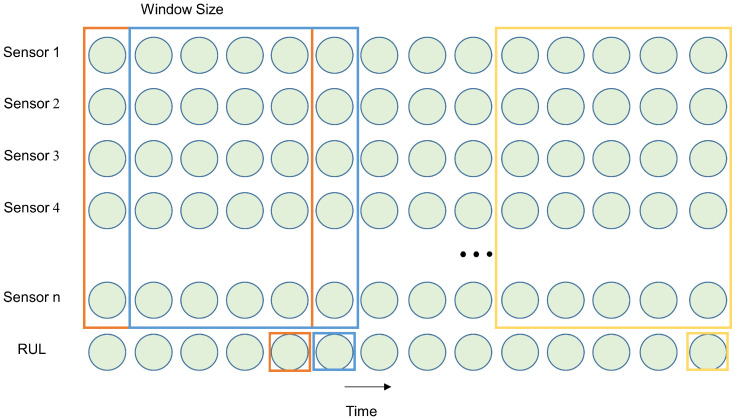
Time windows. The colored boxes represent sliding windows.

The normalized data are segmented into input samples for the TTSNet model through a fixed-width time window in this experiment. The sampling process of the time window is illustrated in Figure 8. In Figure 8, data samples are segmented by moving the window forward one time step along the time axis, and the window slides sequentially until it reaches the end of the data. To capture more detailed information in the time series, the sliding window step size is set to 1 [31]. In the dataset, the RUL corresponding to the last sample data in the window is taken as the remaining useful life label for this window, and each window has one RUL label. After this time window sampling, the input data obtained are a two-dimensional matrix, with the output being the corresponding RUL value.

### 3.5. Setting of RUL Labels

The RUL label for the train set is set as:

(10) $$RUL_{i}^{c}=Cycle_{i}^{\max}-Cycle_{i}^{c}$$

Since the test set engines run from their first operation to a certain point and stop, the data represent an incomplete life cycle. Therefore, the RUL label for the test set is set as:

(11) $$RUL_{i}^{c}=RUL_{i}+Cycle_{i}^{\max}-Cycle_{i}^{c}$$

where $RUL_{i}^{c}$ represents the remaining useful life of the *c*-th sample of the *i*-th model engine, $Cycle_{i}^{max}$ represents the maximum life cycle count of the *i*-th model engine, $Cycle_{i}^{c}$ represents the life cycle of the *c*-th sample of the *i*-th engine, and $RUL_{i}$ represents the life cycle of the *i*-th engine.

**Figure 9:**
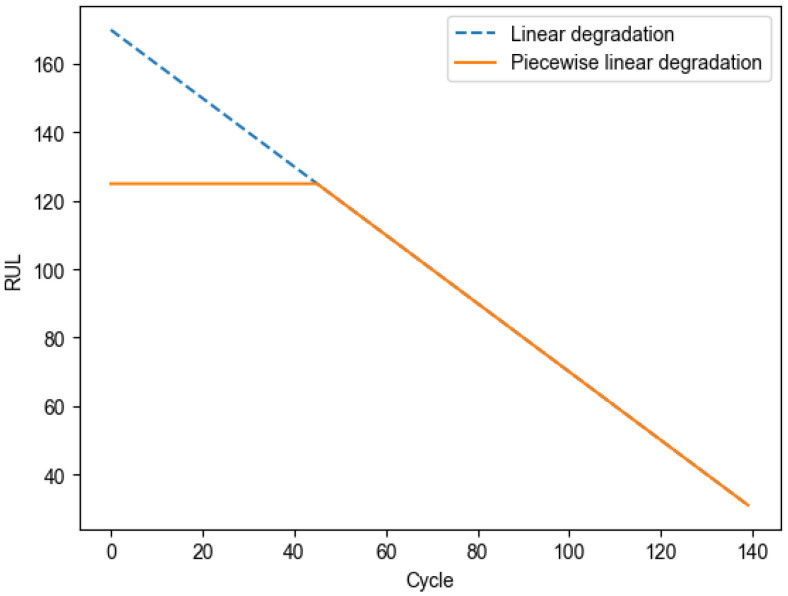
Linear and piecewise linear degradation of aero-engines.

The RUL of engines in the dataset exhibits linear degradation, which makes it difficult for the model to accurately predict RUL and increases the difficulty of training the model. During the initial operating phase, the components of the engine perform well with minimal degradation. As the engine continues to operate, its performance gradually shows a linear degradation trend after the degradation point. Therefore, it is unnecessary to predict the RUL of the aero-engine during its early operating phase. Thus, piecewise linear degradation is used for life prediction in this study. Figure 9 shows the linear and piecewise linear degradation of aero-engines. The piecewise linear degradation formula [9] is as follows:

(12) $$y=\left\{\begin{array}{c} 125,x\geq 125\\ x,x<125 \end{array}\right.$$

where *x* is the linearly degraded RUL, and *y* represents the piecewise degraded RUL value. The remaining useful life at the beginning of the aero-engine’s operation is set to a fixed value of 125, and the actual RUL values greater than or equal to 125 are set to 125, while keeping values less than 125 unchanged. The model enables researchers to effectively predict the remaining useful life of aero-engines by this piecewise linear degradation processing.

## 4. RUL Evaluation Metrics and Experimental Platform

**Figure 10:**
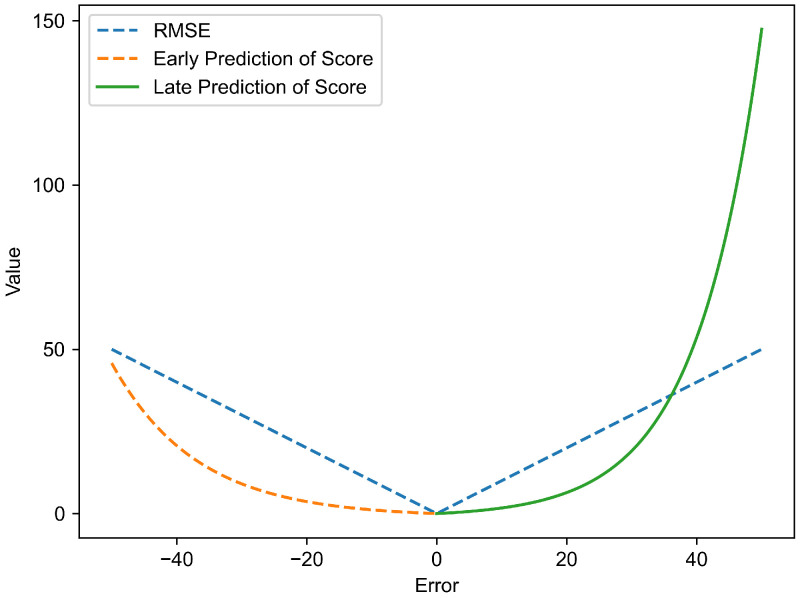
Curve of RMSE and Score.

Root Mean Square Error (RMSE) can represent the average of squared error between predicted and actual values. For the RUL prediction of engines, early predictions are better than late predictions. The score can distinguish between early and late predictions, penalizing late predictions more heavily compared to early predictions [29]. Therefore, to comprehensively evaluate the performance of the RUL model, this study employs two metrics: RMSE and Score. Score is an asymmetric evaluation metric that provides a score based on the difference between the predicted results and the true values, as shown in Figure 10. The horizontal coordinate error in Figure 10 represents the difference between the predicted value and the true value. If the predicted result is less than the true value, it is considered an early prediction, resulting in a lower score; if the predicted result is greater than the true value, it is considered a delayed prediction, resulting in a higher score. The smaller the values of these two metrics, the better the model’s performance.

RMSE is a commonly used performance indicator for prediction tasks, and its formula is as follows:

(13) $$RMSE=\sqrt{\frac{1}{n}\sum_{i=1}^{n}\left[{\left(y_{i}^{pred}-y_{i}^{true}\right)}^{2}\right]}$$

Its formula of score function is as follows:

(14) $$Score=\left\{\begin{array}{c} \sum_{i=1}^{N}\left[e^{\frac{y_{i}-y_{i}^{pred}}{13}}-1\right],y_{i}^{pred}-y_{i}<0\\ \sum_{i=1}^{N}\left[e^{\frac{y_{i}^{pred}-y_{i}}{10}}-1\right],y_{i}^{pred}-y_{i}\geq 0 \end{array}\right.$$

where *n* is the total number of samples, and $y_{i}^{pred}$ and $y_{i}^{true}$ represent the predicted and true values of the ith sample, respectively. *N* is the number of engines.

The experiment was conducted on Linux(Ubuntu22.04) system. The configuration of specific experiment parameters is showcased in Table 3.

## 5. Analysis of Experiment Results

This study uses the Pytorch deep learning framework and employs the Adam optimizer to optimize network parameters. During model training, Mean Squared Error (MSE) is used as the loss function to guide optimization. The learning rate of the optimizer directly affects the training speed and convergence performance of the model. Therefore, to make the network more stable and efficient during training, a linear learning rate adjustment strategy is adopted, with the initial learning rate set to 0.001 and gradually decaying to 0.0001.

Experiments were conducted on FD001–FD004 to test the model’s performance. To obtain suitable hyperparameters, 10-fold cross-validation is performed on the training set. The training set is divided into 10 parts based on the engine ID, and one part is selected as the validation set each time. After 10 experiments, the average RMSE of the validation set is calculated and then the hyperparameters corresponding to the lowest metric are searched for. The hyperparameters of model are set as shown in Table 4. After training the model on the training set, its performance is tested on the test set. To prevent random errors in the experiments, five experiments are conducted under the same conditions for testing.

### 5.1. Impact of Window Size

**Figure 11:**
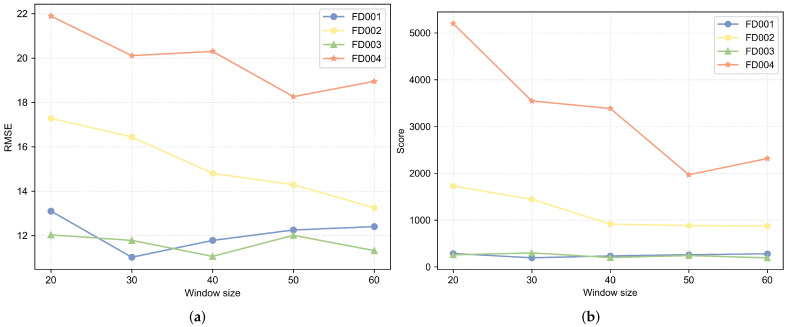
Experimental results of different window sizes on C-MAPSS dataset. (**a**) RMSE of different window sizes. (**b**) Score of different window sizes.

A smaller time window results in fewer degradation information contained in the data samples. A larger time window contains more degradation information. Experiments were conducted to study the impact of different window widths on the model’s predictive capability. In the experiments, the range of the window width was set from 20 to 60, with a step size of 10. The experimental results are shown in Figure 11. As can be seen from Figure 11, in the FD001 dataset, the RMSE and Score are minimized when the window size is 30. In the FD002 dataset, the RMSE and Score are minimized when the window size is 60. In the FD003 dataset, the RMSE and Score are optimal overall when the window size is 40. In the FD004 dataset, the RMSE and Score are minimized when the window size is 50. Compared to single-condition datasets, multi-condition datasets have a larger window width. This is because multi-condition datasets are more complex and the model requires more degradation information.

### 5.2. Comparison Experiments

To verify the effectiveness of the proposed method, the results of the proposed method in this paper were compared with the prediction methods proposed in the existing literature [28,30,31,32,33,34,35,36,37,38]. The comparison results are shown in Table 5. The RMSE of the proposed method is lower than other methods on FD001, FD002, and FD003, and the scores on four subsets are lower than those of DCNN, AGCNN, Attention+TCN, SCTA-LSTM, TATFA-Transformer, and DBA-DCRN methods. On dataset FD001, the RMSE of the proposed method is improved by 0.18% compared to [38], but the Score of the proposed method is higher compared to [30]. On the FD002 dataset, the RMSE of the proposed method improves by 10.1% compared to [30]; the Score of the proposed method is improved by 4.37% compared to [30]. On dataset FD003, the RMSE of the proposed method is improved by 1.5% compared to [37]. On the FD004 dataset, the RMSE of the proposed method improves by 2.9% compared to [37], but its prediction capability is inferior to that of ATCN and MLEAN.

**Figure 12:**
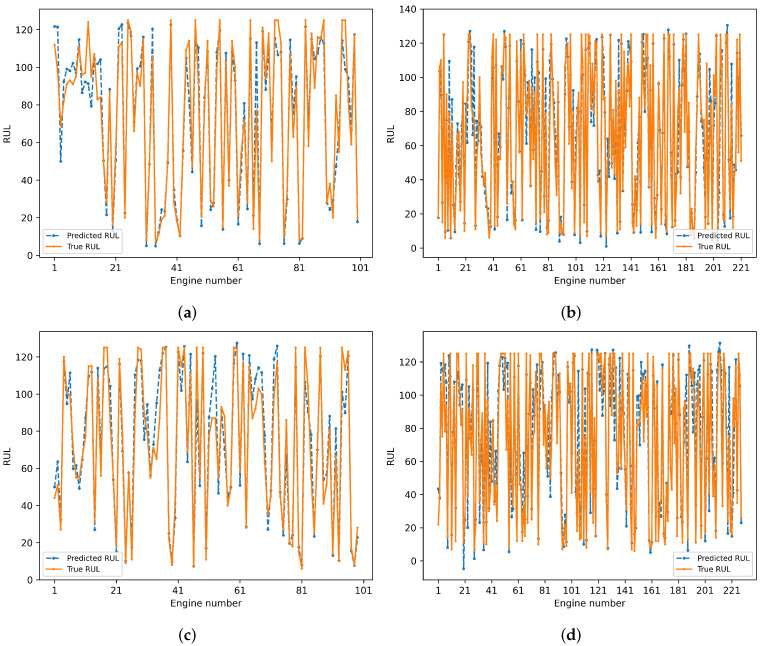
Prediction results of proposed model. (**a**) Prediction results of model on FD001. (**b**) Prediction results of model on FD002. (**c**) Prediction results of model on FD003. (**d**) Prediction results of model on FD004.

Figure 12 shows the comparison of RUL prediction results of the proposed method on the test sets of four subsets. The predicted values of the engines are distributed near the true values, and the prediction curve fits the true curve well. Although the prediction is poor for individual engines, the overall prediction accuracy of the model is high.

**Figure 13:**
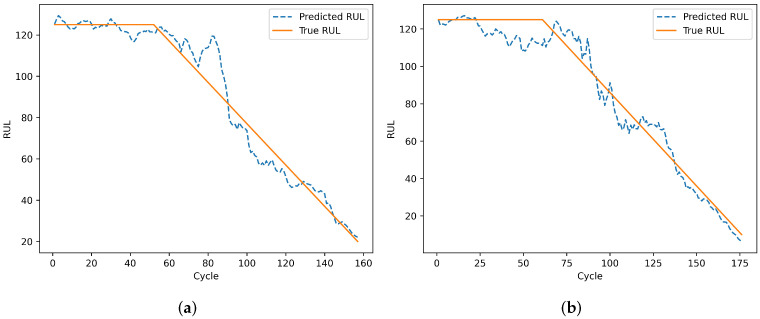
Prediction results of engines. (**a**) Testing engine 24 on FD001. (**b**) Testing engine 76 on FD001.

**Figure 14:**
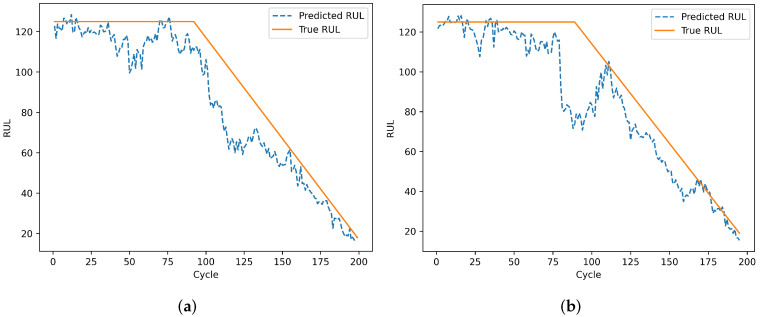
Prediction results of engines. (**a**) Testing engine 1 on FD002. (**b**) Testing engine 185 on FD002.

**Figure 15:**
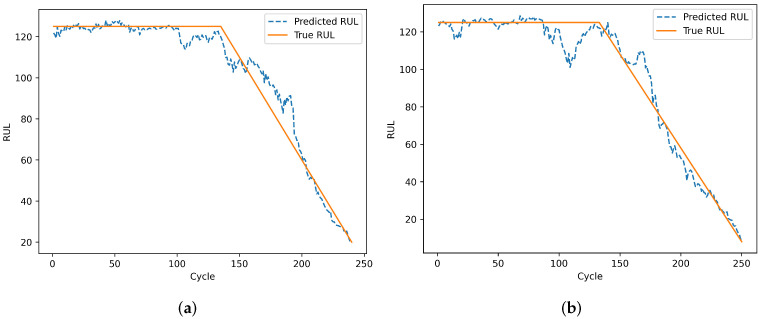
Prediction results of engines. (**a**) Testing engine 78 on FD003. (**b**) Testing engine 99 on FD003.

**Figure 16:**
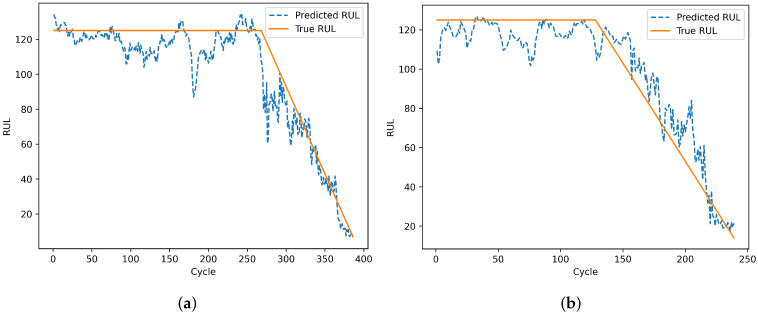
Prediction results of engines. (**a**) Testing engine 135 on FD004. (**b**) Testing engine 213 on FD004.

To evaluate the model’s prediction results on individual engines, engines 24 and 76 from the test set of dataset FD001 and engines 1 and 185 from the test set of dataset FD002 were selected. Engines 78 and 79 from the test set of dataset FD003, and engines 135 and 213 from the test set of dataset FD004 were also selected. The remaining useful life prediction results are shown in Figure 13, Figure 14, Figure 15 and Figure 16. From Figure 13 and Figure 15, although the model prediction curves exhibit some fluctuations, the overall fit between the predicted values and the true values is high. As the number of engine cycles increases and more degradation information accumulates, the model’s prediction results improve. From Figure 14 and Figure 16, compared to FD001 and FD003, the engine prediction curves for FD002 and FD004 show significant fluctuations and poor fitting. The reason is that FD002 and FD004 have multiple operating conditions, making the data more complex, and the model’s extracted features are insufficient. During the degradation phase before a fault occurs, the model’s predicted values are lower than the true operating state values of the engines, enabling the early prediction of the remaining useful life of aero-engines, which has practical predictive value.

### 5.3. Ablation Experiments

To further evaluate the effectiveness of the model designed in this paper, ablation experiments were conducted on four subsets to verify the effectiveness of the ES denoising module, multi-head attention, transformer encoder, and TCN module in RUL prediction. The experimental results are shown in Table 6.

To objectively measure the predictive capability of the model in this study, all prediction models used the same input and fully connected layers. Five models were employed to conduct a comparative analysis of the experimental data. Under the same hyperparameters, the RMSE and Score metrics of the proposed method generally outperformed the other four methods.

The results indicate that the proposed method inherits the strengths of the TCN and transformer models and significantly improves the performance of the RUL model. Comparing the proposed method with the model without TCN, the RMSE and Score of the model on four subsets were better, indicating that the addition of TCN enhances the model’s ability to extract local features. Comparing the proposed method with the model without the transformer encoder, the RMSE and Score on four subsets were better, indicating that the addition of the transformer encoder enhances the model’s ability to extract global features. Comparing the proposed method with the model without the ES module, it was found that the inclusion of the ES denoising module reduced both the RMSE and Score. This result indicates that the ES module can effectively reduce the impact of noise on model prediction. Additionally, when multi-head attention was added, the model’s performance significantly improved. Although the Score deteriorates in FD001, FD002, and FD004, the RMSE decreases in all cases. This demonstrates that self-attention plays a crucial role in distinguishing the contributions of different sensors. In summary, the ES and multi-head attention modules, transformer encoder, and TCN all play important roles in the model of RUL prediction.

## 6. Conclusions

To fully extract features and improve the prediction of aircraft engine remaining useful life, this paper proposes a parallel Transformer-TCN-Self-attention feature fusion network for the RUL prediction of aero-engines. Experiments were conducted on the C-MAPSS dataset, and the results show that using exponential smoothing to process input data can effectively reduce noise; the parallel transformer–TCN network structure can effectively extract global and detailed features from the data; and utilizing the self-attention layer to assign different weights to features can further highlight important features, thereby enhancing the overall performance of the model. The experiments demonstrate that on dataset FD001, the RMSE and Score reached 11.02 and 194.6, respectively; on dataset FD002, the RMSE and Score reached 13.25 and 874.1, respectively; on dataset FD003, the RMSE and Score reached 11.06 and 200.1, respectively; and on dataset FD004, the RMSE and Score reached 18.26 and 1968.5, respectively. This method can accurately predict the remaining useful life of single-condition aero-engines.

This study conducted RUL prediction of aero-engines on the C-MAPSS dataset. Further research plans to use a more powerful Patch time series prediction model than transformer to further improve aero-engine RUL prediction, and to employ transfer learning methods to effectively predict the RUL of aero-engines under complex conditions.

## Figures and Tables

**Table 1:** Overview of C-MAPSS dataset.

Dataset	FD001	FD002	FD003	FD004
Number of training engines	100	260	100	249
Number of testing engines	100	259	100	248
Operation condition	1	6	1	6
Fault mode	1	1	2	2
Total number of training samples	20,631	53,750	24,720	61,249
Total number of testing samples	13,096	33,991	16,596	41,214

**Table 2:** Status monitoring parameters of sensors.

No	Name	Symbol	Unit
S1	Total temperature at fan inlet	T2	K
S2	Total temperature at LPC outlet	T24	K
S3	Total temperature at HPC outlet	T30	K
S4	Total temperature at LPT outlet	T50	K
S5	Pressure at fan inlet	P2	P
S6	Total pressure in bypass-duct	P15	P
S7	Total pressure at HPC outlet	P30	P
S8	Physical fan speed	Nf	r/min
S9	Physical core speed	Nc	r/min
S10	Engine pressure ratio (P50/P2)	epr	-
S11	Static pressure at HPC outlet	Ps30	P
S12	Ratio of fuel flow to Ps30	Phi	-
S13	Corrected fan speed	NRf	r/min
S14	Corrected core speed	NRc	r/min
S15	Bypass ratio	BPR	-
S16	Burner fuel–air ratio	farB	-
S17	Bleed enthalpy	htBleed	-
S18	Demanded fan speed	Nfdmd	r/min
S19	Demanded corrected fan speed	PCNfRdmd	r/min
S20	HPT coolant bleed	W31	lb/s
S21	LPT coolant bleed	W32	lb/s

**Table 3:** Parameters of Experimental environment.

Name	Parameters
Operating system	Linux(Ubuntu22.04)
Processor	Intel(R) Xeon(R) Gold 5128R
Graphics card	RTX3080
Python	3.12.3
Pytorch	2.3.1

**Table 4:** Hyperparameter Setting.

Hyperparameters	FD001,FD003/FD002,FD004
Batch size	64/128
Learning rate	0.001
Epoch	200
Window size	30,40/60,50
Encoder layer	2 layers
	Number of heads on self-attention 2
	Number of feedforward neurons 28
	Dropout 0.2/0.1
TCN Layer	2 layers
	Kernel size 3
	Dropout 0.2/0.1
Linear Layer	Number of neurons 10
Number of heads on multi-head attention layer	5
Number of fully connected layer 1 units	100
Number of fully connected layer 2 units	50
Number of fully connected layer 3 units	1

**Table 5:** Results of comparison experiments. The bold number indicates the best values.

Method	FD001		FD002		FD003		FD004	
	RMSE	Score	RMSE	Score	RMSE	Score	RMSE	Score
DCNN [32]	12.61	273.7	22.36	10,412.0	12.64	284.1	23.31	12,466.0
AGCNN [33]	12.42	225.5	19.43	1492.0	13.39	227.1	21.50	3392.0
Attention+TCN [34]	13.25	235.5	19.37	1655.0	13.43	239.0	21.69	2414.7
Transformer [31]	12.25	198.0	17.08	1575.0	13.39	290.0	19.86	1741.0
MSIDSN [35]	11.74	205.6	18.26	2046.7	12.04	**196.4**	22.48	2910.7
SCTA-LSTM [36]	12.10	207.0	16.90	1267.0	12.14	248.0	21.93	3310.0
ATCN [28]	11.48	194.3	15.82	1210.6	11.34	249.2	17.8	1934.9
MLEAN [30]	11.48	**186.0**	14.74	914.0	11.73	250.0	**16.89**	**1370.0**
TATFA-Transformer [37]	12.21	261.5	15.07	1359.7	11.23	210.2	18.81	2506.4
DBA-DCRN [38]	11.04	201.9	15.31	1084.1	11.81	238.1	18.96	2209.4
Proposed method	**11.02**	194.6	**13.25**	**874.1**	**11.06**	200.1	18.26	1968.5

**Table 6:** Results of ablation experiments. The bold number indicates the best values.

Experimental Method	FD001		FD002		FD003		FD004	
	RMSE	Score	RMSE	Score	RMSE	Score	RMSE	Score
Without ES	11.19	**190.3**	14.17	879.8	11.23	217.9	18.29	2006.3
Without multi-head attention	11.20	194.1	13.38	**534.1**	11.53	235.8	18.43	**1624.8**
Without transformer encoder	11.39	214.3	13.54	1395.6	11.34	217.6	18.27	2136.9
Without TCN	11.53	206.4	15.00	1321.0	11.28	204.9	18.64	2258.7
Proposed method	**11.02**	194.6	**13.25**	874.1	**11.06**	**200.1**	**18.26**	1968.5

## Data Availability

The C-MAPSS dataset presented in the study are available in the Prognostics Data Repository at https://www.nasa.gov/intelligent-systems-division/discovery-and-systems-health/pcoe/pcoe-data-set-repository/ (accessed on 20 November 2024).

## Abbreviations

The following abbreviations are used in this manuscript:

RUL	Remaining Useful Life
TTSNet	Transformer-TCN-Self-attention network
TCN	Temporal Convolutional Network
PHM	Prognostics and Health Management
RNN	Recurrent Neural Network
LSTM	Long Short-Term Memory
GRU	Gated Recurrent Unit
CNN	Convolutional Neural Network
Bi-LSTM	Bidirectional Long Short-Term Memory
RMSE	Root Mean Square Error
